# Role of Sex in Shaping Brain Network Organization During Reading in Developmental Dyslexia

**DOI:** 10.3390/children12020207

**Published:** 2025-02-10

**Authors:** Tihomir Taskov, Juliana Dushanova

**Affiliations:** Institute of Neurobiology, Bulgarian Academy of Sciences, Acad. G. Bonchev Str., Bl. 23, 1113 Sofia, Bulgaria; t.taskov@inb.bas.bg

**Keywords:** functional connectivity, minimum spanning tree model, sex differences, individual differences, developmental dyslexia

## Abstract

**Background/Methods:** The influence of sex on brain organization was investigated in functional reading networks in 8-year-old children, in those typically developing and those with developmental dyslexia (DD), utilizing the minimum spanning tree model. **Results:** The word reading task revealed subtle sex differences in brain connectivity and highlighted even small individual variations in functional connectivity characteristics, particularly among boys with DD. In girls, significantly stronger connections and core hubs were identified within and between motor, parietal, and visual networks in posterior brain regions in both hemispheres, particularly in the θ (dyslexics) and δ (normolexics) frequency bands. In contrast, boys showed a more diffuse connectivity pattern, predominantly in the left hemisphere, encompassing anterior heteromodal and sensorimotor networks. Girls exhibited greater network complexity (bigger leaf fraction, kappa, and tree hierarchy), particularly in the θ and δ frequency bands, while boys with DD showed increased network efficiency, except for in the γ2 band (smaller diameter and bigger leaf fraction). Therefore, gender-specific differences in brain network organization may affect reading development and dyslexia. While sex may influence brain network development, its impact on the sensorimotor and frontoparietal networks of 8-year-old children is relatively limited. Significant sex differences were observed in only a small subset of children, primarily in higher (β2-γ2) frequency bands. **Conclusions:** Interindividual variations were evident only in boys with DD, impacting both sensorimotor and association networks. Different rates of cortical network maturation between sexes with DD during childhood may contribute to variations associated with disruptions in brain network development, even within fundamental networks like the sensorimotor network.

## 1. Introduction

Reading involves two primary pathways that rely on distinct brain regions [1,2,3]. The sublexical route (dorsal occipitoparietal) converted letters to sounds (graphemes to phonemes), which is crucial for unfamiliar words [2]. The lexical route (ventral occipitotemporal) recognizes familiar words rapidly, which is essential for skilled reading and responsible for whole-word recognition [3]. Early reading instruction focuses on grapheme–phoneme correspondence, learning how letters or groups of letters represent sounds, and phonological decoding, applying these rules to decipher unfamiliar words. This foundational skill is crucial for reading comprehension. As children progress, they transition from explicit instruction to self-learning, developing the ability to decode words automatically.

Children with developmental dyslexia experience phonological deficits, difficulties with identifying and manipulating sounds, challenges with the sublexical route, and problems with grapheme–phoneme conversion [3]. Dyslexia can involve phonological and visual deficits and cognitive processing inefficiencies. The phonological deficits are related to difficulties with sound awareness and processing, while visual deficits are related to issues with letter perception and spatial organization (e.g., letter reversals). Any children with dyslexia, who have difficulty with phonological awareness and effectively connecting sounds to letters, have deficits in the ability to decode words and their processing in the associative decoding network [4]. Visual–spatial deficits are related to poor letter perception, such as letter reversals and difficulty with spatial organization [5]. Cognitive processing inefficiencies are related to difficulties with efficient information processing, potentially due to distractions or interference [6]. Reading development varies significantly among individuals and is influenced by differences in vocabulary, phonological skills, and orthographic knowledge [4]. Understanding the interplay of these factors is crucial for developing effective interventions. Individual differences in vocabulary, phonology, and orthographic skills significantly impact reading development.

Reading is a complex cognitive process that engages multiple brain regions and functions, including visual, phonological, orthographic, and semantic systems [7]. The occipital cortex, supramarginal gyrus, fusiform gyrus, and anterior temporal cortex are the main brain regions associated with these systems. Furthermore, the frontal opercular and temporal cortices play crucial roles in reading. While these brain regions are directly involved in reading, the auditory cortex also contributes significantly to reading achievement, likely due to the strong link between phonological awareness and successful reading acquisition [7]. Reading aloud involves significant activity in the left motor and somatosensory cortices, as evidenced by studies on speech production [8]. Visual processing, crucial for recognizing written words, engages visual areas of the brain [1]. Additionally, language-related regions, particularly semantic areas such as the inferior frontal, inferior temporal, and superior temporal cortices, are integral components of the neural networks underlying reading comprehension [9]. While reading is predominantly left-lateralized, involving main areas like Broca’s area (for phonological processing) and the left fusiform gyrus (for orthographic processing) [9,10,11], recent research has demonstrated the significant involvement of the right hemisphere in reading [12]. The involvement of right hemisphere regions, such as the prefrontal and posterior temporal areas in language comprehension has been shown to impact reading skills [13]. Strong functional connections within the parietal and frontal cortices of both hemispheres, crucial for higher-order cognitive functions, are consistently observed in reading networks. Reading networks involve a complex interplay between both hemispheres, with each hemisphere playing a specialized role. Studies have shown that the two hemispheres may process different frequency ranges of the acoustic speech envelope during reading [14]. Individual differences in reading ability are influenced by the right hemisphere’s contribution to lexical processing [15].

Analyzing the patterns of connectivity between different brain regions provides insights into how the brain processes information [16]. Concepts in brain network analysis include functional segregation, which refers to specialized processing within specific brain regions, and functional integration, which describes the ability of different brain regions to communicate and share information [16]. By studying these principles, we can gain a deeper understanding of how brain networks support reading and how they are affected by dyslexia. These concepts, often studied using graph methods, help to understand how the brain processes information. Brain networks exhibit “small-world” properties characterized by both strong local connections and efficient long-distance connections. This efficient organization undergoes significant reorganization during development, transitioning from a more random to a more segregated and specialized network [17].

Developmental dyslexia (DD) is a complex learning disorder that may manifest differently in women and men. Recent research suggests that the neurological underpinnings of dyslexia could vary between sexes [18]. This finding has significant implications for our current understanding of dyslexia and the development of effective strategies to compensate for reading disabilities. Recent studies highlight the importance of considering sex differences in dyslexia research [19]. By examining the differential neuroanatomical profiles between women and men, we can gain valuable insights into the brain mechanisms underlying this disorder. Research on the interplay between biology, behavior, and the brain has produced inconsistent findings when considering sex differences. While both biological and social factors influence these differences, the relationship is intricate. Genetic studies are crucial in identifying sex differences by examining genetic predispositions linked to each sex.

The research on sex differences in the adolescent brain’s individual network is inconclusive. While factors like genes, hormones, and environment influence brain morphology, the extent to which these factors lead to significant sex differences in network organization remains unclear. However, studies have shown that certain brain networks, such as the ventral and dorsal attention networks and frontoparietal networks, are more effective in distinguishing sex in young individuals [20]. This is likely due to the impact of sex hormones on the connectivity within these networks [20]. These networks are linked to mood and fear symptoms, and research suggests that females may exhibit greater abnormalities in connectivity related to fear symptoms within specific networks [20,21].

Motor and sensory networks are relatively consistent across individuals, while significant variability is observed particularly in associative networks like the default mode and ventral attention networks [20]. Compared to sensorimotor networks, this variability is highest in the group map similarity of functional topology in associative networks [20]. Regions within the frontoparietal network also show a high degree of interindividual heterogeneity. This variability in the functional topology of association cortices is linked to individual differences in cognitive abilities. While sex alone cannot fully account for individual differences, research has shown normative sex differences in the functional topology of adolescent associative networks. This suggests that sex partially influences interindividual differences in functional topology. Individual differences in cognitive abilities during childhood can impact physical, social, and mental outcomes in adulthood.

Research shows how the brain’s functional networks change as children aged 9 and 10 grow [19,22] by studying the topology of these networks and individual differences in cognitive abilities. The study found that the frontoparietal and ventral attentional network variations are the strongest predictors of these differences, while the visual and somatomotor networks have a weaker predictive power. Understanding these variations is crucial as it can help us better understand the transition to adolescence.

Our previous research found differences in the brain network functions between typically developing children and those with developmental dyslexia (DD) [23]. Using graph theory (the minimum spanning tree method), this electroencephalogram (EEG) study showed that children with DD have abnormal brain network connections in the local and global organization of brain networks involved in reading between typically developing children and those with developmental dyslexia, and they constructed reading networks in multiple EEG frequency bands. Compared to typically developing children, children with DD had a less organized (less segregated) brain network, as evidenced by higher leaf fraction, tree hierarchy, and kappa values, and a smaller network diameter (θ/γ frequency bands). Additionally, dyslexic children exhibited reduced connectivity (reduced degree and betweenness centrality of hubs) in brain regions, including the superior, middle, and inferior frontal areas, in both hemispheres, especially in the β1 and γ-frequency networks. While typically developing children relied on a more balanced network involving various brain regions, children with DD seemed to compensate for this by relying more heavily on specific areas, such as the left anterior temporal (β1, γ1) and dorsolateral prefrontal cortex (γ1), as well as the right hemisphere’s occipitotemporal, parietal (β1), motor (β1, γ1), and somatosensory cortices (γ1).

The present study aims to investigate how these brain networks vary among individual children with DD and their typically developing peers, focusing on differences between boys and girls aged 8–9. Using word reading difficulties, we hope to gain insights into specific problems by analyzing how certain brain areas become less active (deactivated) or how connections between them change. Another unanswered question in research on dyslexia is how the strong connections within brain networks change during specific word reading and how these changes affect overall brain function. Understanding these changes, especially concerning sex and individual differences, is crucial.

Given the ongoing development of the central nervous system and the age-related changes in brain function, even in typically developing children, it is crucial to understand the relationship between age and frequency functional networks (delta, theta, and alpha EEG rhythms) strongly linked to brain maturation. Additionally, we need to explore the specific developmental networks of reading abilities. In children with learning disabilities, high theta (θ) and low alpha (α) activity often indicate delayed development of cognitive brain networks [24,25]. However, the impact of increased cognitive effort on the brain activity of stimulus-related brain regions during challenging tasks remains unclear. Behavioral assessments may not be as sensitive as needed to understand the achievement of fully automated multisensory integration [26]. Therefore, there is a growing interest in developing new methods to modify brain networks and improve reading abilities. The main research points included new insights into the neurological basis of dyslexia that can be gained through brain network analysis, applying specific reading tasks that influence the activity of brain networks at specific ages of children; the contribution of variations in the strength and connectivity between brain regions (hubs) to dyslexia; and linking the functional reading network to the specific oscillatory (frequency) activity of some brain regions (hubs).

This study aimed to investigate the functional connectivity of the brain in children with dyslexia using graph theory through minimum spanning tree analysis. Our goals were to (1) provide an objective interpretation of the findings; (2) demonstrate the potential and limitations of these techniques; and (3) determine if there are significant sex differences in brain networks in control children and in those with dyslexia, and if these differences are associated with an individual- or group-specific experimental paradigm.

We hypothesized the following: (1) compared to normolexics, children with DD exhibit different “minimum spanning tree” and different strong connections within various frequency sub-networks, which may vary between sexes; (2) personalized frequency functional networks within separate sex subgroups during the experimental word reading task will remain stable if they are not associated with age-related brain organization; and (3) changes in specific brain regions (hubs) within certain frequency networks will be linked to the brain’s functional network during reading, particularly if the functional networks of children with dyslexia differ from those of normolexics.

## 2. Materials and Methods

### 2.1. EEG Data Acquisition and Processing

A 40-channel wireless EEG system [27] with star-shaped dry gold sensors was used to record electroencephalographic (EEG) signals with a sampling rate of 250 Hz. Electrodes were positioned according to the 10-10 and 10-20 systems (10/10: AF3-AF4, F7-F8, FT9-F10, FC3-FC4, FC5-FC6, C1-C2, C5-C6, CP1-CP2, CP3-CP4, TP7-TP8, P7-P8, PO3-PO4, PO7-PO8: Fz, F3-F4, C3-C4, Cz, T7-T8, P3-P4, Pz, O1-O2, Oz; [28,29]), with reference sensors on the processus mastoidei and ground electrodes placed on the forehead. Skin impedance was kept below 5 kΩ to ensure signal quality. Individual trials were segmented into 800 ms windows with stimulus onset. EEG signals were filtered (bandpass: 1–70 Hz; cutoff filter for 50 Hz) and artifact rejections were above ±200 µV. To evaluate the quality of the signal, the signal-to-noise ratio (SNR) was calculated according to the following formula: SNR = A/(2 × SDnoise) [30]. The amplitude A was the peak-to-peak potential of the mean ERP, and the SD of the noise (ε) was obtained by subtracting the mean from each ERP. Only trials with good SNR and correct responses were included in the analysis. The frequency bands in which the EEG transforms were filtered were δ(1.5–4), θ(4–8), α(8–13), β1(13–20), β2(20–30), γ1(30– 48), and γ2(52–70) Hz.

### 2.2. Application of a Minimum Spanning Tree Method to Experimental Design

Some authors have used a simplified representation of the original network, called a minimum spanning tree (MST), to compare different brain networks [16]. Networks can be represented as either binary or weighted graphs. Binary connections indicate the presence or absence of a connection, while weighted connections provide information about the strength of the connection. The weights represent the densities or efficiencies of connections in the weighted network [16]. Weighted networks can be simplified by removing weak or unimportant connections. These connections can obscure the underlying topology of strong and significant connections. A threshold can be applied to the weights to eliminate these weaker connections. Before analysis, self-connections and negative connections, such as functional anticorrelations, should be removed from the networks. The phase lag index (PLI) was used to calculate the correlation between each pair of EEG channels, resulting in an adjacency matrix [31]. Weighted adjacency matrices (40 × 40) were constructed based on the weighted PLI (wPLI) for all pairs of sensors evaluated across the δ- and γ-frequency networks. Each wPLI matrix was used to construct a separate MST sub-graph from the non-rejected realizations. An MST is a unique sub-graph that connects all nodes without forming loops, minimizing the weights of the connections (the cost of “wiring”). The finite MST topologies represent three null models: a line-like topology, a star-like topology, and an intermediate hierarchical tree [31]. MST does not capture most of the properties of a complex network, particularly in a scale-free sub-network. Scale-free networks are characterized by a power-law distribution of connections to nodes, where most nodes have few connections, and a few important nodes (hubs) have many connections. Due to acyclicity, the resulting networks have a lower density, which can lead to a loss of information about the original network [32]. This approach was employed to investigate complex brain networks in the current study, focusing on dyslexia-related deficits in the strong connections of the functional reading network. By quantifying the topological properties of network representations, this approach aims to elucidate important characteristics of functional brain networks. Various network measures can characterize global and local brain connectivity. Global measures quantify functional integration and segregation [16,33] and assess the resilience of networks to external perturbations.

In this study, the global measures of MST used were diameter, leaf fraction, tree hierarchy, and kappa. The diameter of the MST (the shortest path in the minimum spanning tree) measures the efficiency of the network’s global organization.

The leaf fraction LF = L/m is the number of leaves L, defined by the number of nodes with only one link, within the MST, relative to the total number of nodes m. When the LF is high, communication largely depends on the hubs from which the leaves extend.

The tree hierarchy (TH) characterizes the putative optimal topology of efficient organization while preventing information overload on the central nodes. TH = L/(2mBC_max_) is determined by the number of leaves L, nodes, and links m in the MST, and the maximum betweenness centrality BCmax of MST, where the betweenness centrality for each node is calculated as the portion of shortest paths that go through it.

The diameter D = d/m is a measure of network efficiency that refers to the largest distance (d), as the number of links m, between any two nodes of the tree, normalized by the total number of links.

The degree refers to the number of connections for a given node i, k_i_ = ∑_*j∈N*_(a_ij_), where *N* is the number of nodes. The weighted version of the degree is defined as the total sum of the weights of all neighboring connections. The degrees of all nodes in a network have a distribution that is a marker of the development and stability of the network. Degree values reflect the importance of nodes in the network.

Kappa (K) characterizes the homogeneity of the distribution of the number of links of the MST. Higher K values reflect a border degree distribution (degree divergence ҡ = ‹*k*^2^›/‹*k*›) and greater vulnerability to targeted impacts. All global measures of MST were calculated separately for each non-rejected implementation of the reading task.

The local measures of nodes (node measures) used in the study, such as degree and betweenness centrality (BC), provide information about the importance of individual nodes in the network [33,34,35]. The BC is defined by the number *p_h__j_* of shortest paths between any two nodes *h* and *j* in the network and the number *p*^(i)^*_hj_* of shortest paths between them (*h*, *j*) that pass through a given node i divided by the total number of shortest paths, such that BC_i_= (∑*_h_*_,*j∈N*, *h*≠*j*, *h*≠*i*, *j*≠*i*_ *p*^(i)^*_hj_*/*p_hj_*)/((*n* − 1)(*n* − 2)).

Nodes with a degree or high BC are considered “hub nodes”; this is not based on the number of their connections but on their importance to global communication in the network. They play an important role in information processing in the network [33].

Networks are more integrated when they have a higher maximum degree or maximum BC [35]. Hubs were defined as nodes with a degree or BC exceeding the average node strength by at least one standard deviation [34]. Connections were considered significant if their weight exceeded the mean weight of all connections by at least one standard deviation. The networks are strongly influenced by the network’s global properties, such as the number of nodes, connections, and the distribution of node degrees. These measures were computed for both control and children with DD during a reading task to identify changes in the strongest connections within the reading network. Individual brain networks were characterized using a combination of brain imaging techniques, anatomical parcellation methods, and connectivity measures, employing the MATLAB BrainNet Viewer 1.7 functional package (http://www.nitrc.org/projects/bnv/ (accessed on 31 October 2019) [36]). It visualizes brain networks by mapping nodes and edges to specific brain regions, assigning them sizes and colors based on their defined properties, and incorporating topological features of the brain.

### 2.3. Reading Task

The study used a controlled visual presentation of single words to examine reading behavior and brain activity in children (described in detail in [23]). Single words were presented as visual stimuli. These words were chosen to be age-appropriate and varied according to their frequency of use, and the focus was on 2–3 syllable words that were 6–8 letters long. The words were displayed on a 1920 × 1080-pixel screen at a refresh rate of 60 Hz for 800 ms. The font used was Microsoft Sans Serif, and the words were presented in a pseudo-random sequence. Each word was presented at a distance of 57 cm from the observer. The font size of each letter was 1 degree of visual angle, and the spacing between letters was one-third of the letter width.

Participants underwent daily EEG sessions. Stimuli were presented in blocks of 40 words. Participants were instructed to read each word aloud. To minimize artifacts in the EEG data, they were asked to avoid blinking during the word presentation and only blink during the inter-stimulus interval (1.5–2.5 s). The steep initial and final slopes of the audio signal waveform for each word determined the duration of word reading. The following reading-related behaviors were measured:

(1) Vocal Reaction Time: The time elapsed from the onset of the word on the screen to the initiation of reading.
(2) Word Reading Duration: The total time spent reading each word.
(3) Reading Speed: The number of words read correctly during the task.

### 2.4. Statistical Analysis

Non-parametric tests were used because the data did not follow a normal distribution. Permutation tests were used to assess the significance of observed differences by randomly permuting the data and comparing the observed test statistics to a distribution of test statistics obtained under the null hypothesis. Non-parametric Kruskal–Wallis tests were used to compare the behavioral parameters between children with developmental dyslexia (DD) and typically developing children.

Non-parametric bootstrap tests with 1000 random permutations were used to compare global MST measures of D, LF, TH, and K between the sex subgroups for each frequency network [37]. A Bonferroni correction was applied to control for multiple comparisons (*p* = α/4 = 0.0125).

Cluster-based permutation tests were used to identify clusters of nodes in the brain network that showed significant differences between groups. Non-parametric cluster-based permutation tests were applied to compare local MST measures (hD and hBC) between sex subgroups and performed so that hBC_max_/degree_max_ crossed a selective threshold criterion for defining a hub. When multiple clusters show a significant difference, the effect is measured according to their rank. Their indices in the histograms are chosen so that their medians are sensitive to interhemispheric differences. These tests identify clusters of hubs with significant differences. A Bonferroni correction was applied to control for multiple comparisons on the threshold (*p* = α/2 = 0.025). The same selection criteria were performed separately, and the relationships crossing the threshold are represented in red in the figures.

### 2.5. Selection of Groups

Children participated in the EEG study after their parents signed informed consent forms according to the Declaration of Helsinki. The Ethics Committee of the Institute of Neurobiology approved the study (No. 41/12-07-2019). A neuropsychological examination was conducted [38]. A standardized test battery, DDE-2, was utilized to assess developmental dyslexia in children, specifically identifying various deficits related to reading and writing [39,40]. The Girolami-Boulinier test assesses nonverbal perception using the “Differently Oriented Signs” exercise [41,42]. All participants took a nonverbal intelligence test using Raven’s Progressive Matrices [43]. Psychometric tests were used to assess phonological awareness, reading, and writing in primary school children using a test battery for evaluating written language skills [44]. The DD group and the control group differed in both the number of correct answers and the time taken to complete the tests (F(2, 47) = 31.4, *p* < 0.05; ANOVA; Table S1). All the children examined with developmental dyslexia displayed a normal IQ on the Raven test, which is consistent with the score expected for their respective ages (F(2, 47) = 1.8, *p* = 0.76). The group with DD consisted of 24 participants (12 boys and 12 girls), with a mean age of 8.6 years (±0.4). The normolexic group included 24 participants (12 boys and 12 girls), with a mean age of 8.44 years (±0.6). Both groups were second graders from families with average socioeconomic status. The study included right-handed native Bulgarian-speaking children [45] with no reported neurological or psychological disorders and normal or corrected-to-normal vision.

## 3. Results

### 3.1. Between-Group Parameters in Word Reading Task

Comparisons between groups revealed that the experimental group (boys and girls with DD) exhibited slower vocal reaction times (vRTs), longer reading durations (dRTs), reduced reading speeds, a lower success rate, and more omitted words compared to the control group (control) (χ^2^ ≥ 36.24, *p* < 0.0001; Kruskal–Wallis test, Table 1).

Significant differences were found in all global MST measures between controls and the dyslexic group in the θ, β, and γ networks, as well as for local measures in the γ-frequency networks for word reading task, as described in our previous work ([23]; Table 1 and Table S2).

### 3.2. Within-Group Design of Behavioral Parameters in Word Reading Task

**Figure 1:**
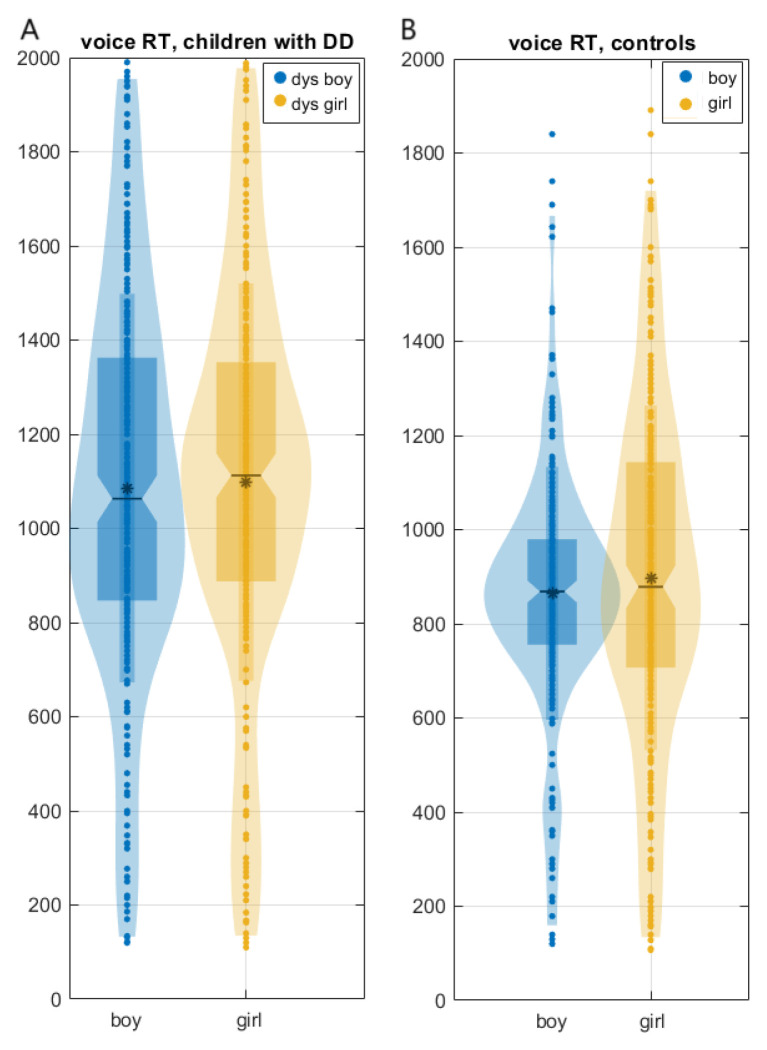
Vocal reaction times (median, -; mean, *) of boys (blue) vs. girls (yellow) in the reading task: (**A**) boys vs. girls in the group with DD; (**B**) boys vs. girls in the control group.

When reading words, boys with DD showed statistical differences (Figure 1; Table S2) compared to girls with DD in the following tasks:

(1) Vocal reaction times were significantly shorter in boys with DD (median (CI): boys with DD: 1045.7 (1035.39, 1109.18) ms; girls with DD: 1110 (1075.64, 1155.06) ms (χ^2^ = 6.17; *p* = 0.01)).

(2) The time to read a correctly pronounced word was shorter in girls with DD (boys with DD, median (CI): 753.7 (733.23, 789.17) ms; girls with DD: 671.23 (631.13, 711.33) ms (χ^2^ = 5.34, *p* = 0.0021)).

There was no statistical difference in reading speed between boys with DD and girls with DD.

There was no statistical difference in vocal reaction times and reading speed between boys and girls in the control group: boys (median, CI) 848 (830.01, 872.56) ms; girls 855.4 (845.02, 914.34) (χ^2^ < 0.102, *p* > 0.74, Figure 1).

### 3.3. Within-Group Comparison of Global MST Measures of Functional Connectivity

**Figure 2:**
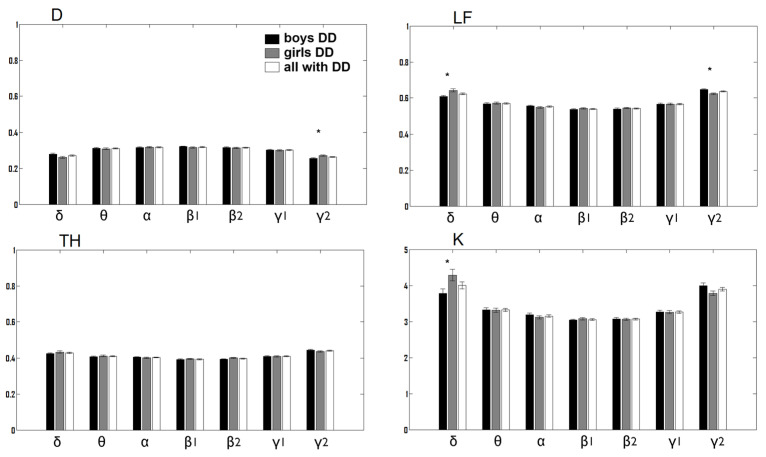
Global measures (D, LF, TH, K, mean ± s.e.; *p* < 0.01 *) of the frequency networks for boys with DD (black color), girls with DD (gray color), and all children with DD (white columns) in a reading task.

**Figure 3:**
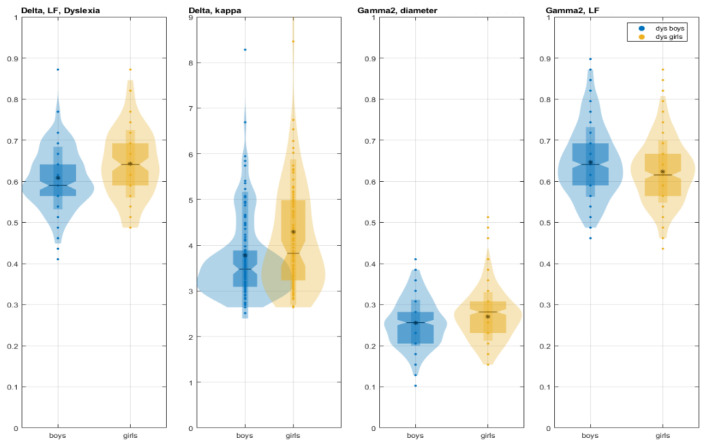
Significantly different global measures of LF and K (median, -; mean, *) of the δ-frequency network and D and LF of the γ2-frequency network between boys with DD (blue) and girls with DD (yellow) in the reading task.

In word reading, the girls with DD showed significantly larger global measures than the boys with DD (Figure 2 and Figure 3). This was observed in the δ network at LF (χ^2^ = 10.59, *p* = 0.001) and K (χ^2^ = 8.87, *p* = 0.003), as well as in the γ2 network at D (χ^2^ = 9.67, *p* = 0.002). Conversely, the girls exhibited a smaller LF (χ^2^ = 8.96, *p* = 0.003) in the γ2 network.

**Figure 4:**
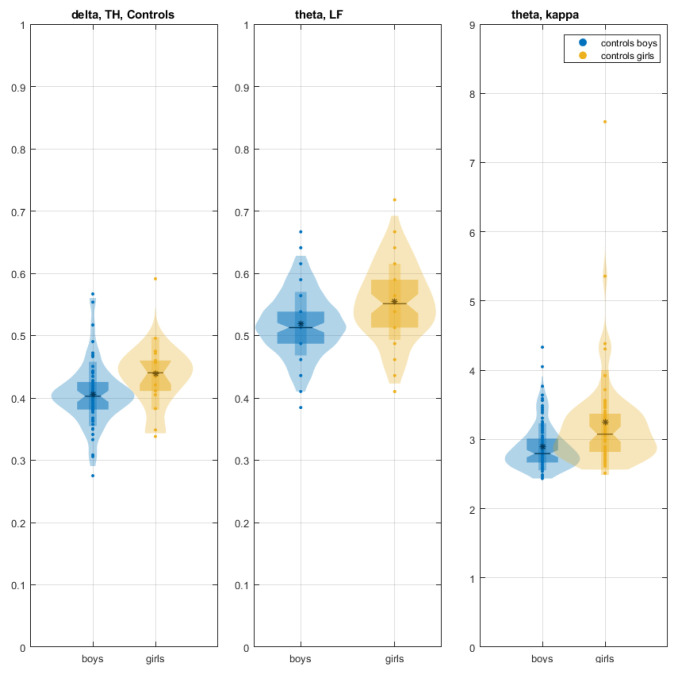
Significantly different global measures of TH (median, -; mean, *; δ-frequency network, 1st plot) as well as LF and K (median, -; mean, *; θ-frequency network, 2nd and 3rd plot) between boys (blue) and girls (yellow color) in the control group during word reading.

The TH of the δ network was greater for control girls than for control boys in word reading (χ^2^ = 6.498, *p* = 0.0107), as were LF of the θ network (χ^2^ = 13.95, *p* = 0.0002) and kappa (χ^2^ = 19.53, *p* = 9.89 × 10^−6^) (Figure 4).

### 3.4. Within-Group Comparison of Local Measures of Frequency Networks

#### 3.4.1. Sex Differences in Group with DD

**Figure 5:**
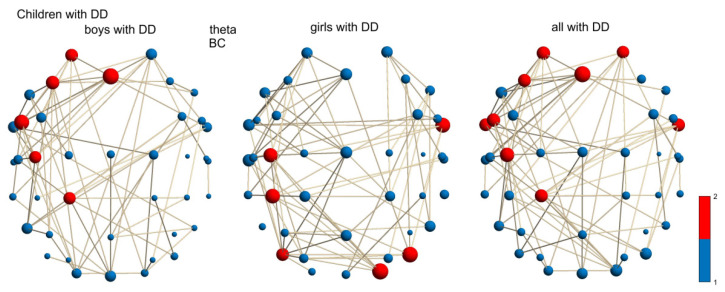
Theta-frequency network in boys with DD, girls with DD, and mixture group with DD (girls and boys with DD): BC of hubs of θ network for boys: AF3, Fz, F3, FC5, C3, CP1; girls: FT10, C3, CP3, PO7-PO8, O2; and all children with DD: AF3-AF4, F3, Fz, FT9-FT10, FC5, C3, CP1.

The median of the hub distribution of girls with DD was statistically larger than that of boys with DD in the θ-frequency network (according to hub BC, χ^2^ = 4.992 and *p* = 0.025; Figure 5). The hub distribution for boys with DD was in the left hemisphere, covering superior frontal (AF3), middle frontal (Fz, F3), inferior frontal (FC5), postcentral (C3), and superior parietal (CP1) areas sensitive to eye movements, language comprehension, expression, reading, visuomotor, visuospatial attention, and memory processing. The hubs with the highest BCs were in both hemispheres of the girls with DD at the anterior part of the inferior temporal (FT10), primary somatosensory and motor (C3), inferior parietal (CP3), and middle occipital (PO7-PO8, O2) cortices related to language comprehension, visual integration, and visual attention processing. The group with all DD children had hubs in both hemispheres at superior frontal (AF3-AF4), middle frontal (Fz, F3), inferior frontal (FC5), anterior part of the inferior temporal (FT9-FT10), postcentral (C3), and superior parietal cortices (CP1) associated with eye movements, language comprehension, expression, speech, reading, visuomotor, visuospatial attention, and memory processing.

Girls and boys with DD showed hubs in the left primary motor cortex and non-hub nodes in the left posterior middle temporal and superior temporal (Wernicke’s area) areas. Separately, boys with DD showed hubs in the left superior parietal cortex and left middle frontal lobe (compared to controls; Figure S1). Girls with DD had overactivation in the right middle occipital region and non-hub nodes in the left inferior and superior frontal cortices. The left occipitotemporal region was found only in control boys and was absent in all children with DD and control girls.

#### 3.4.2. Sex Differences in Control Group

**Figure 6:**
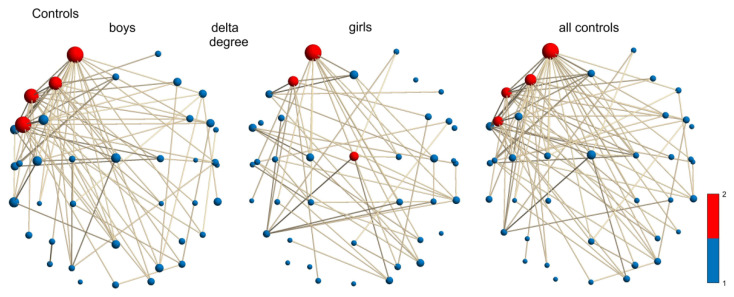
Delta-frequency network (degree of hubs) of control boys, control girls, and mixture group (control girls and boys): boys: AF3, F3, FC5, F7; girls: AF3, F3, Cz; all (control girls and boys): AF3, F3, FC5, F7.

In the δ network (according to the hub degree, χ^2^ = 5.03 and *p* = 0.0249; Figure 6), the control boys had a larger median of the differentially distributed hubs in the left-hemispheric superior frontal (AF3), middle frontal (F3), and inferior frontal (FC5, F7) areas related to the frontal eye fields, as well as language and reading areas, compared to the hub distribution in the control girls for language comprehension, expression, and reading processing areas, which are mostly in the left hemisphere at superior frontal (AF3), middle frontal (F3), primary motor, and premotor cortices (Cz). All controls had hubs in the left-hemispheric superior, middle, and inferior frontal cortices (AF3, F3, FC5, and F7) related to the saccadic movements, language expression, speech, and visuospatial memory.

The δ-frequency network (degree of hubs) of control boys had hubs in the left inferior frontal cortex compared to control girls. This was a normal activity (normal maturation) because it also existed in the group with all the children. The δ-frequency network of control girls had hubs in the primary motor and premotor cortices, which were not found in the control mixture group. Therefore, there was no overactivity, as in the boys with DD, girls with DD, and the mixture group with DD (see Figure S2).

### 3.5. Exploring Interindividual Variation in Functional Networks

Individual differences in the 9–10-year-olds’ brain functional networks are linked to variations in their cognitive abilities. According to the model linking brain networks to cognitive performance [22,46], specific brain networks along the somatosensory axis, particularly those involved in sensory processing, contribute to cognitive functions. To explore this further, we examined individual differences in global and local brain activity patterns in 8-year-olds.

**Figure 7:**
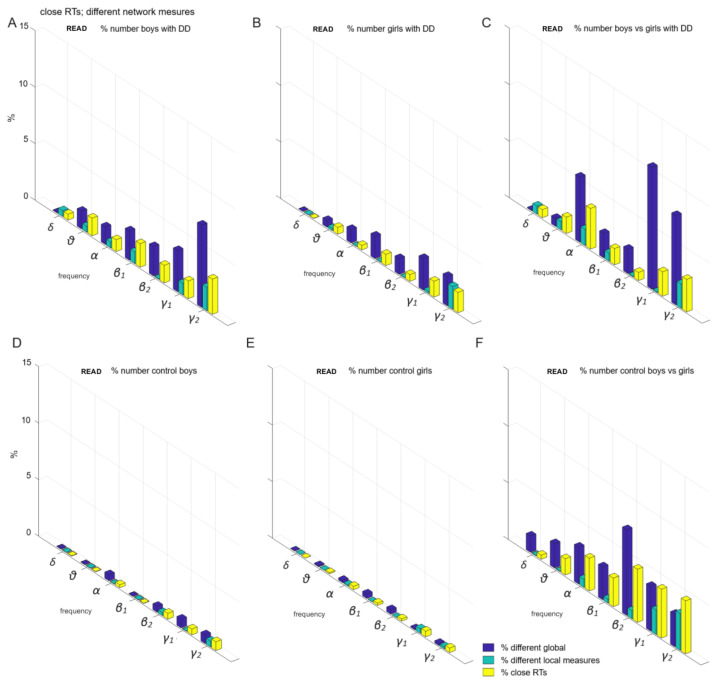
Individual variations in global and local measures for participants with similar reaction times (*p* ≥ 0.05) during a word reading task: (**A**) boys with DD; (**B**) girls with DD; (**C**) boys vs. girls with DD; (**D**) control boys; (**E**) control girls; (**F**) all control participants (boys and girls).

(1) When children exhibited similar reaction times, suggesting that sensorimotor networks were not significantly contributing to the task (*p* ≥ 0.05, Figure 7), we investigated the role of frontoparietal networks. We examined the following subgroups:

(a) Boys with DD, representing up to 7% of the total sample (Figure 7A).
(b) Girls with DD, accounting for up to 3% of the sample (Figure 7B).
(c) The comparison between boys and girls with DD, showing a difference of up to 11% (Figure 7C).
(d) Control boys, representing 1% of the total sample (Figure 7D).
(e) Control girls, also representing 1% of the total sample (Figure 7E).
(f) The comparison between control boys and girls, showing a difference of up to 8% (Figure 7F).

(2) When we considered the case of the sensorimotor’s equal contribution in both sex groups, we then concluded that the frontoparietal and associative networks still remain underdeveloped in all sex subgroups. Associative networks, in particular, have not yet undergone the necessary plasticity to adapt to environmental influences, experiences, or interventions designed to enhance cognitive development.

**Figure 8:**
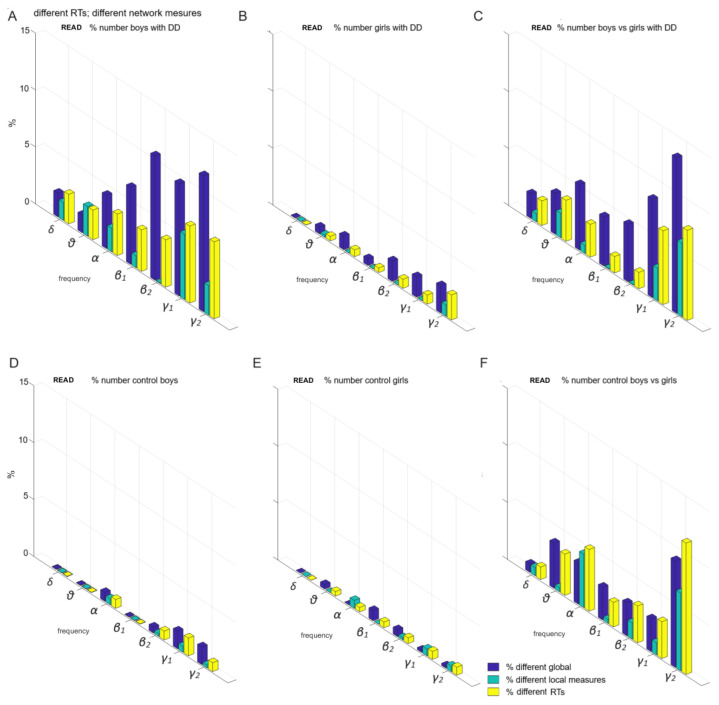
Individual variations in global and local measures with different reaction times (*p* ≤ 0.05) in reading: (**A**) a subgroup of boys with DD; (**B**) girls with DD; (**C**) boys vs. girls with DD; (**D**) control boys; (**E**) control girls; (**F**) all control participants (boys vs. girls).

The sensorimotor network contributed to individual differences in global and local measures in children with significantly different reaction times (*p* ≤ 0.05, Figure 8).

This was evident in the following subgroups:

(a) Boys diagnosed with DD, representing up to 12% of the total sample (Figure 8A).
(b) Girls with DD, accounting for 2% of the sample (Figure 8B).
(c) The comparison between boys and girls with DD, showing a difference of up to 13% (Figure 8C).
(d) Control boys, representing 2% of the total sample (Figure 8D).
(e) Control girls, accounting for 1% of the total sample (Figure 8E).
(f) The comparison between control boys and girls, showing a difference of up to 9% (Figure 8F).

Sex differences during brain development, particularly in developmental dyslexia, may be linked to variations in sensorimotor networks. Children with DD, especially boys, often show delayed development of sensorimotor networks, which likely contributes to their greater individual variability. At this age, both associative and sensorimotor networks are still maturing, limiting the emergence of significant individual sex-based differences.

## 4. Discussion

Sex is a biological factor that influences the organization of the brain, leading to differences in the structure and function of cortical and subcortical networks [47]. These variations may underlie sex differences in cognitive abilities, socioemotional skills, executive functions, and psychopathology at a specific age, which are potentially linked to genetic factors [20,21]. However, existing studies have primarily examined sex differences in brain topology at the group level, limiting our understanding of the relationship between brain structure and gene expression at the individual level [20,21].

The sensorimotor association axis, which spans from the unimodal visual and somatomotor cortices to the transmodal association cortex, exhibits the greatest variability in functional network organization supporting cognitive abilities [22]. Individual differences in the organization of associative networks may be critical for predicting variations in cognitive abilities, as different networks likely contribute to cognitive function in diverse ways. Certain brain regions maintain a high degree of plasticity for an extended period, making them more susceptible to environmental influences and personal experiences. This contributes to the unique patterns observed among individuals. Moreover, the prolonged plasticity of associative networks suggests that targeted interventions could effectively promote cognitive development.

In the present study, we investigated global and local functional brain connectivity measures in relation to sex differences. We found a substantial overlap in the organization of association networks between boys and girls, with a difference of only 8% of all comparisons (Figure 7F). However, the data suggest that sex differences at the age of 8 are more strongly associated with variations in sensorimotor, visual, and control networks (up to 11%; Figure 8F). These findings indicate that sex differences at this age are primarily related to individual differences in functional connectivity, suggesting that these differences in developmental dyslexia appear to be more closely related to specific brain deficits.

### 4.1. Relationship Between Variability in Network Connectivity During Reading and Development of Control Group

Compared to control boys, control girls exhibited greater LF and K in the θ network, suggesting greater communication on the hubs from which the leaves extend and increased vulnerability to targeted disruptions. Additionally, the higher tree hierarchy TH in the δ network of girls indicated a less optimal network topology with a potential for information overload on central nodes. Girls demonstrated a greater functional integration within the δ and θ networks, likely enabling them to combine information from diverse brain regions [24]. In boys, the δ and θ networks were more segregated than in girls. During brain development, increased segregation of brain functional systems plays a crucial role in optimizing brain network organization. This enhanced segregation is characterized by greater specialization of function within specific brain regions and reduced functional overlap between them. In typically developing children, increased brain system segregation is associated with superior cognitive abilities, such as strong long-term episodic memory [48]. Brain networks with well-segregated systems exhibit greater flexibility in the face of certain types of interference. The θ-frequency neural network for reading predominantly involves frontotemporoparietal connections within the left hemisphere (Figure S1; [49]). This network is initiated by early visual processing in the right superior occipital gyrus and the inferior occipitotemporal cortex [50], which act as start hubs for transmitting information throughout the reading network [51]. Subsequent stages involve the bilateral postcentral gyri (motor and somatosensory cortices), the left inferior frontal cortex, and the dorsolateral prefrontal cortex [49]. These regions are crucial for processing lexical and sublexical phonological information and play a significant role in articulatory processes essential for reading [52,53,54,55,56,57]. The θ reading network also includes the left superior parietal, right postcentral, bilateral motor, and premotor areas, and the right primary somatosensory cortex. These regions contribute to the articulatory processes involved in reading [52]. Control boys exhibited stronger functional connectivity within their θ reading network, compared to girls, between the posterior inferior temporal cortex (adjacent to the posterior fusiform gyrus), a crucial area for word processing, and the visual and cognitive regions [58]. This enhanced connectivity may be associated with the increased time spent reading by control boys [58]. Reading engages multiple brain regions and functions, including visual, phonological, orthographic, and semantic systems. These systems are represented by the occipital cortex, supramarginal gyrus, fusiform gyrus, and anterior temporal cortex. The θ network of control boys exhibited a distinct hub, adjacent to Heschl’s sulcus, that was not observed in girls. The simultaneous presence of hubs in the left inferior frontal lobe and middle temporal lobe (adjacent to the visual word-form area), observed in control boys but not in girls (θ network; Figure S1), may be associated with reading development [59].

In the control group, variations in the local functional θ-frequency network were found within and between motor networks (up to 4%, Figure 8F), and to a lesser extent within and between frontoparietal and associative networks (Figure 7F) [60]. Regarding the functional β2-frequency network variations, the control subgroups exhibited the most variations within and between frontoparietal and associative networks (up to 7%, Figure 7F). Sex functional differences in α and γ2 networks also involved connections within and between frontoparietal and associative networks, affecting up to 3% of children (Figure 7F).

The local γ2-network variations in the control subgroups included variations in the global measures within and between motor networks and involved variations in the degrees and BCs of hubs within and between them (up to 11%, Figure 8F). The functional θ-, α-, and γ2-network differences within and between motor networks were attributed to a sex difference within the control group (Figure 8F).

### 4.2. Relationship Between Variability in Network Connectivity During Reading and DD Deficits

Girls with DD exhibited greater leaf fraction and kappa in the δ-frequency network compared to boys with DD, indicating greater reliance on hub nodes and increased vulnerability to disruptions within this network, which may lead to information overload within central hubs, potentially hindering optimal global network organization. Conversely, girls with DD showed a greater diameter and smaller leaf fraction in the γ2 network, potentially reflecting higher segregation, decreasing the load on the important brain regions, and leading to a more balanced brain network, analogous to the processes observed in the brain development of children. The subgroups showed different levels of development in these frequency networks. Boys with DD demonstrated a smaller diameter and higher leaf fraction in the γ2-frequency network, suggesting a more integrated, “star-like” network compared to girls with a more decentralized network. However, the boys with DD had optimal global network organization in the δ-frequency network (smaller leaf fraction and kappa). These observed differences in network topology across subgroups likely reflect distinct compensatory mechanisms. These findings align with evidence and theoretical models suggesting deficits in general sensory functions and attention in dyslexia, which are often associated with higher frequency EEG activity (beta and gamma) [61]. Reduced segregation within task-related brain systems in dyslexia is often observed initially, but this tendency subsides with practice and task automation as dyslexic individuals with higher levels of network segregation may exhibit greater cognitive improvements with training [23]. Functional abnormalities in the θ network of girls with DD were characterized by the absence of hubs in the frontal lobes and the presence of hubs in the anterior temporal lobe (right hemisphere) and the inferior parietal and middle occipital regions (left hemisphere). During word reading in girls with DD, early visual processing within the θ-frequency network was restricted to the bilateral occipital cortex, serving as the starting point for major hubs that transmit information to the left-hemispheric premotor and supplementary motor cortices [62]. In contrast, boys with DD exhibited an absence of hubs in visual brain areas within the θ network. Crucially, several main hub areas, including the occipital, middle, and superior temporal cortices, as well as the parietal/occipital cortex near the angular gyrus, which are typically specialized in the left hemisphere during reading skill acquisition, were absent in boys with DD [49]. The absence of hubs adjacent to Heschl’s sulcus in children with DD (theta), a characteristic observed in control boys, aligns with findings from other studies that identify this absence as an early indicator of dyslexia [63]. This research supports the hypothesis that phonological decoding deficits in dyslexia, affecting both visual and auditory processing, can be positively influenced by visual training [64,65]. Furthermore, the findings emphasize the involvement of both heteromodal and modality-specific brain areas in the complex process of reading.

The local θ-network variation within motor networks among subgroups with DD was primarily attributable to differences in the BC of hubs between sexes (Figure 5) and partially to differences in global measures of the LF and kappa of motor networks (Figure 3 and Figure 8C).

Network differences between subgroups with DD at γ-frequency networks (Figure 8C) were largely influenced by sex differences in the connections between hubs with the highest degrees or BCs within and between motor networks in 7% of boys with DD (Figure 8A; green color). The differences in network connectivity among subgroups with DD in β- and γ-frequency networks were primarily attributed to sex differences in global measures within and between motor networks in 13% of children with DD (Figure 8C; blue color). They were influenced by individual variations within and between these networks among the boys with DD, as noted in 11% of them (Figure 8A).

In contrast, the local α- and γ1-network differences among subgroups with DD were mainly attributed to variations in global measures of frontoparietal and associative networks (12%, Figure 7C). In the other frequency networks, differences between the DD subgroups largely stemmed from individual variations in global measures within/between frontoparietal and association networks in up to 7% of boys with DD (Figure 7A).

### 4.3. Relationship Between Variability in Network Connectivity and Sex

A variability in global functional connectivity was observed in associative networks among girls and boys for both groups (Figure 7C,F), but a greater variability in local functional connectivity was found in motor networks among boys and girls within both controls and children with DD (Figure 8), as reported by Long et al. 2017 [60].

These findings indicate that sex might be a more complex construct that is not as clearly represented in functional connectivity and may not yet be fully reflected in the functional cortical network of 8-year-old children. The lack of significant associations between sex and functional connectivity could be attributed to the limited variability in these measures, which may arise from the immature development of cortical areas at the age of 8 or the relatively small sample size of children (DD and controls [46]). It is not surprising that the proportion of internal variation in boys with DD (unrelated to sex, but rather related to different maturation) was relatively small (12%). In girls, it was even lower (1%). Our findings indicate that there are associations between functional connectivity and sex, suggesting that the sex of 8-year-old children influences the organization of brain networks in a minority of children because functional brain networks mature unevenly in cortical networks across genders during childhood [65].

Unimodal sensory networks, which respond to stimuli within a single sensory modality, mature first, followed by heteromodal associative networks involved in cognitive processes. All these networks contribute to local functional sex differences. This study also found that the relationship between sex and connectivity within and between sensorimotor, visual, and motor networks was stronger than those in frontoparietal and associative networks. In girls, the strongest connections and core hubs were found within and between motor, parietal, and visual networks located primarily in posterior regions in the theta (dyslexics) and delta (normolexics) frequency bands. In contrast, boys showed a more diffuse pattern of connectivity, encompassing heteromodal and sensorimotor networks, predominantly anteriorly in the left hemisphere. Sex differences in functional capacities between association and sensorimotor cortices are partly due to regional differences in interhemispheric functional connectivity profiles. These differences may arise from differential refinement of connectivity during brain development, and this variability differs across the cortical hierarchy [47].

Sex manifestation in children undergoes significant changes during puberty, which is marked by structural and functional brain maturation [66] (9–10-year-old children). Childhood development before puberty, brain maturation, and their relationship to reading disorders subtly influence individual and sex-specific differences in the sensitivity of the reading brain network.

Understanding this variation in child brain development can help capture the biological variability associated with the emerging psychopathology of dyslexia. This suggests that studying brain changes in early childhood is essential for identifying relevant biological variations in the processes that shape brain development. Furthermore, findings related to psychopathological associations show sensitivity to subtle manifestations of psychopathological problems that may not yet have reached clinical thresholds. This approach may provide insights into additional differences in behavioral or mental health measures. Brain maturation and sex differences introduce additional variability in the results for reading abilities.

## 5. Conclusions

We found that reading tasks magnify subtle individual differences in immature brain functional connectivity among 8-year-olds, predicting developmental issues and reading disabilities in a sex-specific way. Our small sample, similar to Luo et al. [47], revealed regional variations in functional connectivity likely influenced by developmental maturation [22]. These findings align with previous research [67] suggesting that developmental differences in functional connectivity may underlie variations in reading abilities. While sensory–motor networks become more integrated, associative networks remain more segregated during childhood [47]. This differential development might explain the variability in functional connectivity observed between boys and girls with developmental dyslexia (DD). Moreover, boys with DD show atypical development of functional connectivity, even within sensorimotor networks, deviating from the expected age-related maturation.

## Figures and Tables

**Table 1:** A statistical comparison of behavioral parameters between the experimental and control groups.

	DD	Control	KW Test
	Mean ± s.e.	Mean ± s.e.	*p*, *x*^2^
vRT [ms]	1093.8 ± 19.48	865.48 ± 14.22	<0.0001, 376.4
dRT [ms]	734.46 ± 26.76	579.39 ± 30.21	<0.0001, 250.5
Speed [N/s]	21.74 ± 2.28	39.65 ± 3.07	<0.0001, 30.78
Success [%]	70.74 ± 3.7	99.32% ± 0.41	<0.0001, 36.24
Omitted words [N/s]	12 ± 1.5	<1 ± 0.16	<0.0001, 36.24

## Data Availability

The data are not publicly available due to the restrictions applied to the availability of the data. The data presented in this study are available on request from the corresponding author.

## Supplementary Materials

The following supporting information can be downloaded at https://www.mdpi.com/article/10.3390/children12020207/s1: Figure S1: θ-frequency network in control boys, girls, and mixture group (girls and boys): (1) hubs with highest BC for boys: AF4, F7, C5, P7, T8, Oz; girls: AF3, FT9-FT10, Fz, Oz, O1; all controls: Fz, F7, FT9, TP7, Oz, O1. Figure S2: δ-frequency network in boys with DD, girls with DD, and mixture group (girls and boys with DD): (1) hubs with large degree for boys with DD: AF3, F7, F3, C3; girls with DD: AF3, F7, F3, C3; all with DD: AF3, F7, F3, C3. Table S1: Psychological scores of the groups. Table S2: comparisons of the means of behavioral parameters. We cited the [38,39,40,41,42,43,44,45] in the Supplementary Materials.
