# Supplementary material for: Role of Sex in Shaping Brain Network Organization During Reading in Developmental Dyslexia
Source: Children (Basel). 2025 Feb 10;12(2):207. doi: 10.3390/children12020207 (PMC11854611; doi:10.3390/children12020207)
Supplement: Supplementary file 1 [file children-12-00207-s001.zip › Supp_Tables_S1_S2_corect.pdf]

Role of Sex in Shaping Brain Network Organization During Reading in Developmental Dyslexia

Tihomir Taskov and Juliana Dushanova *

Institute of Neurobiology, Bulgarian Academy of Sciences, Acad. G. Bonchev Str., bl. 23, 1113 Sofia, Bulgaria; t.taskov@inb.bas.bg
* Correspondence: juliana@bio.bas.bg

Table S1. Psychological scores of the groups in standard scores (mean±s.d.; 2nd and 5th column). The statistics (F, p) of boys with DD vs girls with DD (3rd, 4rt column); control boys vs girls (6th, 7 columns); statistics of controls vs group with DD (8th and 9 columns).

1. DDE-2 test	DD mean±s.d.	DD boys/girls F(1,23)	DD boys/girls p	controls mean±s.d.	control boys/girls F(1,23)	control boys/girls p	Controls/DD F(1,47)	Controls/DD p
1.1. Reading words								
accuracy	90.6±4.5	0.74	0.41	106±5.58	0.24	0.71	**5.53**	**0.03**
time (s)	91.7±4.8	0.6	0.47	132±0.76	0.15	0.85	**11.6**	**0.003**
1.2.Reading pseudowords								
accuracy	88.3±4.4	1.03	0.33	102±4.65	0.73	0.53	**8.45**	**0.01**
time (s)	96.7±4.1	0.71	0.41	118±0.68	0.41	0.82	**7.06**	**0.01**
1.3 Homonyms (accuracy)	98.5±2.4	1.65	0.22	112±4.82	1.65	0.22	**4.44**	**0.04**
1.4 Spelling (accuracy)	112±4.45	0.001	0.9	112±1.82	0.13	0.86	0.22	0.804
1.5 Writing words (accuracy)	85.7±4.4	2.76	0.12	115±6.49	1.88	0.32	**5.1**	**0.03**
1.6 Writing pseudowords (accuracy)	91.9±3.9	2.76	0.12	104±4.25	1.06	0.52	**6.31**	**0.02**
1.7 Dictation (accuracy)	89.5±3.4	3.27	0.1	112±4.82	2.07	0.1	**5.21**	**0.04**
2. Psychometric tests	DD mean±s.d.			Controls mean ± s.d.				
2.1. Reading text								
correct answers	119.8±7.9	0.73	0.40	129.4±3.4	0.41	0.63	**4.92**	**0.03**
време (s)	191.56±7.9	0.61	0.51	104.8±29.0	0.79	0.82	**8.37**	**0.008**
2.2. Dictation								
Correct sentences	10.9±4.51	1.753	0.201	21.0±5.9	1.05	0.23	**7.19**	**0.01**
2.3 Phological task Without the first sound								
accuracy	5.5±2.04	0.01	0.96	9.20±1.9	0.22	0.65	**6.35**	**0.02**
time (s)	62.5±29.4	0.31	0.85	34.9±10.6	0.12	0.89	**5.9**	**0.03**
2.4 Phological task Without the last syllable								
correct answers	6.2 ± 2.4	2.36	0.11	8.05±2.1	2.36	0.11	**8.50**	**0.006**
time (s)	64.8±30.5	2.4	0.10	37.50±8.8	1.04	0.53	**9.23**	**0.04**
3. Girolami-Bulinier								
correct answers	40±2.1	2.3	0.11	50.9±9.3	2.26	0.11	6.19	0.014
4. Raven's test	102±4	0.44	0.85	108±9	1.12	0.41	1.42	0.86

Developmental Dyslexic Group (DD): 24 children (12 boys, 12 girls), mean age 8.6 ± 0.4 years.
Control Group (Normolexics): 24 children (12 boys, 12 girls), mean age 8.44 ± 0.6 years.
All participants were second-graders from families with average socioeconomic status. They had normal or corrected-to-normal vision and were right-handed native Bulgarian speakers.
Inclusion Criteria: All children underwent a comprehensive neuropsychological screening [38] to ensure eligibility for the study. All participants in the study spoke Bulgarian as their first language and were right-handed [45]. The children had normal or corrected-to-normal vision after examination by an ophthalmologist. The dyslexic group comprised children with reading difficulties, characterized by below-average performance (more than one standard deviation below the mean of age-matched standardized controls) in reading speed or accuracy on the DDE-2 battery and the

"Reading Abilities" test. The control group consisted of age-matched children from the same socio-demographic background as the dyslexic group. These children demonstrated typical reading performance, with within-norm scores in reading speed and accuracy on both the DDE-2 and "Reading Abilities" tests. No child in the control group had a diagnosis of dyslexia or co-occurring language disorders.

Assessments:

Cognitive Abilities:

DDE-2: A standardized battery for evaluating Developmental Dyslexia and Dysorthography in Bulgarian children [39,40], assessing reading (word and nonword reading, homonym recognition, misspelling detection) and writing (word and nonword dictation, sentence dictation).The reading included 112 words/48 nonwords with increasing complexity; 10 homonyms—choosing the correct meaning of the word from 4 variants; searching for misspellings of 32 words. The writing included dictation of 47 words/26 nonwords with increasing complexity; 12 sentences. The results are presented in standard reading time/speed and accuracy scores.

Reading Abilities Test or Bulgarian children [44]**:** Assessed phonological awareness (identifying and omitting the first sound/last syllable of words), reading fluency (reading a text aloud), and writing (dictation of sentences). The test battery “Reading abilities" comprised two phonological tasks, each with 10 words; reading aloud a text with 133 words and dictation of 30 sentences filling in the missing compound word.

Phonological Awareness:

Two tasks were administered:

"Without the first sound-letter": Children identified and then omitted the first sound of the heard word in his/her answer.

"Without the last syllable": Children fragmented the word into syllables and then omitted the last syllable of the word in his/her answer.

Performance was measured by accuracy and execution time.

The Girolami-Boulinier test [41,42] assessed nonverbal perception using the “Differently Oriented Signs” exercise.

Raven's Progressive Matrices Test [43]: Assessed nonverbal intelligence.

Children with DD exhibited significantly lower scores on all assessments compared to the control group.

38. Raichev, P.; Geleva, T.; Valcheva, M.; Rasheva, M.; Raicheva, M. Protocol on Neurological and Neuropsychological Studies of Children with Specific Learning Disabilities. In *Integrated Learning and Resource Teacher*; Evgenieva, E., Ed.; Publishing House “Dr. Ivan Bogorov”: Sofia, Bulgaria, 2005; pp. 82–105. (In Bulgarian).
39. Matanova, V.; Todorova, E. *DDE-2 Test Battery for Evaluation of Dyslexia of Development—Bulgarian Adaptation*; OS Bulgaria Ltd.: Sofia, Bulgaria, 2013; Available online: https://www.giuntipsy.bg/bg/prod-19-testova-bateriq-za-ocenka-nadisleksiq-na-razvitieto.htm (accessed on 1 October 2013).
40. Sartori, G.; Remo, J.; Tressoldi, P.E. Updated and Revised Edition for the Evaluation of Dyslexia. In *DDE-2, Battery for the Developmental Dyslexia and Evolutionary Disorders-2, 1995*; Giunti, O.S.: Florence, Italy, 2007.
41. Girolami-Boulinier, A. *Contrôle des Aptitudes à la Lecture et à l'Ecriture (CALE)*; CALE: Paris, France, 1985. (In French)
42. Yakimova, R. *Abnormalities of Written Speech*; Rommel Publuling House: Sofia, Bulgaria, 2004. (In Bulgarian)
43. Raven, J.; Raven, J.C.; Court, J.H. Manual for Raven's Progressive Matrices and Vocabulary Scales. In *The Colored Progressive. Patrices*; Oxford Psychologists Press: Oxford, UK; The Psychological Corporation: San Antonio, TX, USA, 1998.
44. Kalonkina, A.; Lalova, Y. *Normative Indicators for the Test Battery for a Written Speech Assessment*; Iossifova, R., Ed.; Rommel Publishing House: Sofia, Bulgaria, 2016; pp. 30–38.
45. Annett, A. A classification of hand preference by association analysis. *Br. J. Psychol.* **1970**, *61*, 303–321.

Table S2. Statistical comparisons of the means of behavioral parameters (p, χ2, Kruskal–Wallis test; significance level, $p < 0.05$) in reading.

	**DD boys**	**DD girls**	*p*	χ^2	**Contro boys**	**Control girls**	*p*	χ^2
	mean ± s.e.	mean ± s.e.			mean ± s.e.	mean ± s.e.		
vRT [ms]	1048.4±8.21	1108±6.33	**0.01**	**6.17**	848.01±5.24	858.4±4.6	0.42	0.64
dRT [ms]	753.7±6.76	671.23±5.35	**0.002**	**5.34**	567.32±30.71	582.39±18.31	0.12	0.75
Speed [N/s]	22.63±2.3	20.85±2.5	0.063	4.9	39.85±3.2	38.94±2.17	0.1	0.78
Success [%]	69.82±3.6	71.65±2.7	0.69	0.2	97.86±0.32	99.4±0.51	0.85	0.03
Omitted words [N/s]	13±1.4	11±1.8	0.79	0.1	<1 ± 0.16	<1 ± 0.23	0.9	0.0004
