# Supplementary figures and images for: Role of Sex in Shaping Brain Network Organization During Reading in Developmental Dyslexia
Source: Children (Basel). 2025 Feb 10;12(2):207. doi: 10.3390/children12020207 (PMC11854611; doi:10.3390/children12020207)

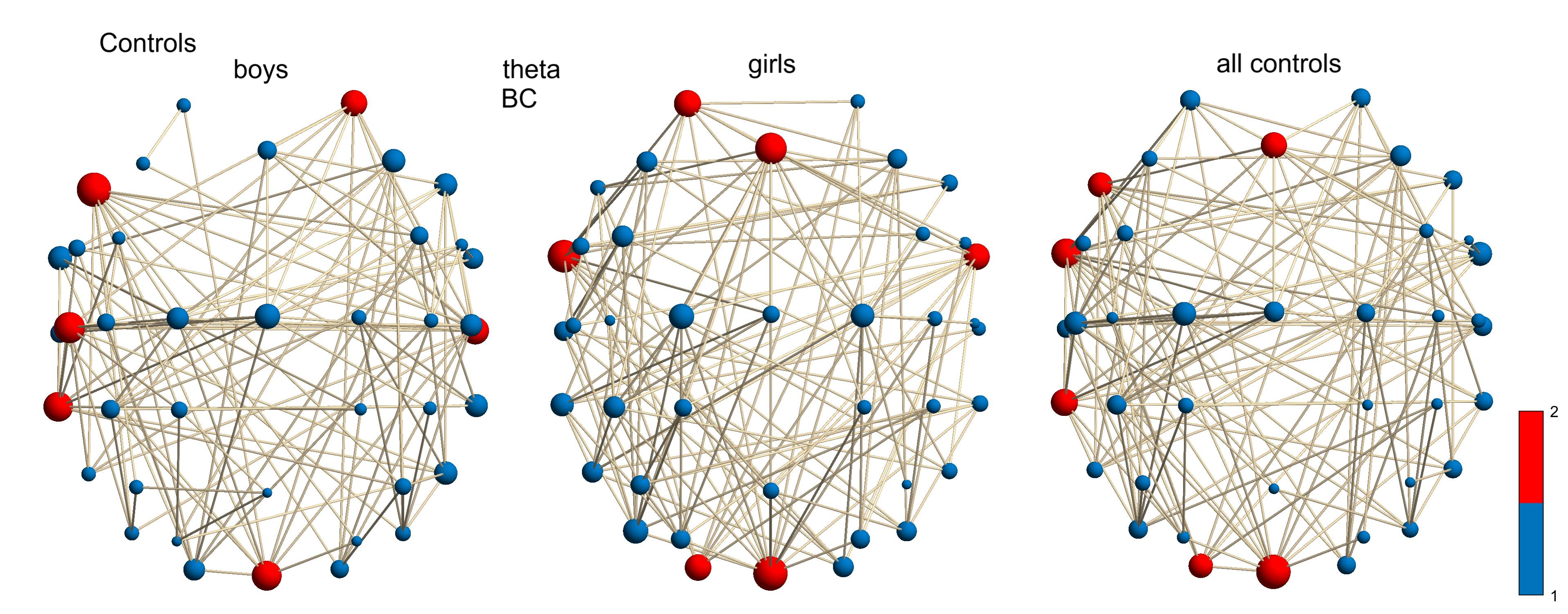

Supplement: Supplementary file 1 [file children-12-00207-s001.zip › Figure S1_контроли_boys_girls_all mst_BC_theta_stim1.bmp]

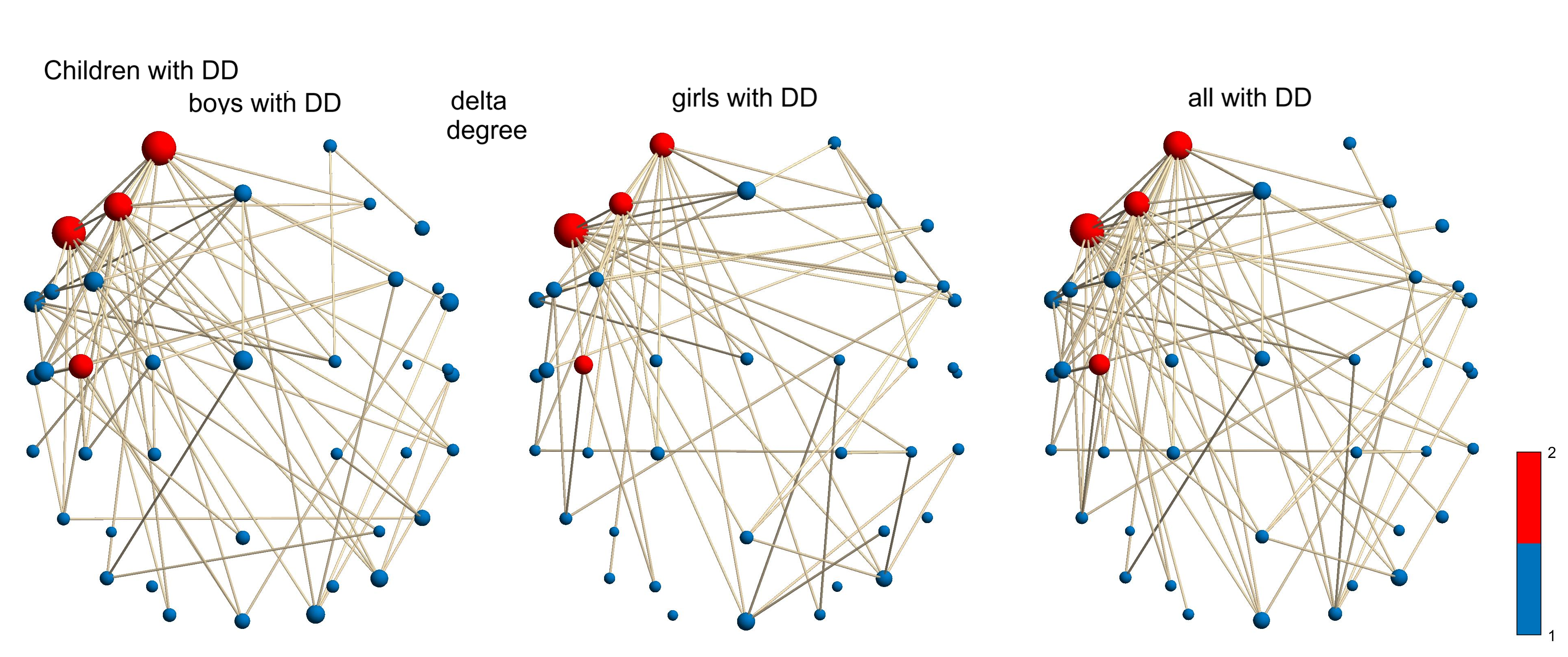

Supplement: Supplementary file 1 [file children-12-00207-s001.zip › Fig_S2_dys_boys_girls_mst_degree_delta_stim1.bmp]
